# Recurrent Phyllodes Tumour of the Breast Transforming to a Fibrosarcoma

**DOI:** 10.7759/cureus.7457

**Published:** 2020-03-29

**Authors:** Dua Jabeen, Lubna M Vohra, Tariq Siddiqui, Aziz-un-nisa Raza

**Affiliations:** 1 Medicine, Jinnah Sindh Medical University, Karachi, PAK; 2 Breast Surgery, Aga Khan University Hospital, Karachi, PAK; 3 Oncology, Ziauddin University Hospital, Karachi, PAK; 4 Pathology, Ziauddin University Hospital, Karachi, PAK

**Keywords:** phyllodes tumour, fibrosarcoma, breast

## Abstract

Phyllodes tumours of the breast are characterized by having both an epithelial as well as stromal component and these usually comprise almost 3% of all fibroepithelial tumours. They are exceptional in this aspect to convert into a stromal sarcoma of the breast after multiple recurrences. To the best of our knowledge, there are only three case reports regarding this in the available literature as it is an exceptional change. In this case report, we present a case of recurrent phyllodes transforming into a breast fibrosarcoma in a middle age postmenopausal woman. Histopathological examination and immunohistochemistry of the lesion were performed to confirm the diagnosis of breast fibrosarcoma.

## Introduction

Phyllodes tumours or biphasic tumours can be identified by its leaf-like architecture due to its intra-canalicular growth pattern, cleft-like spaces lined by epithelium, and hyper cellular stroma [1]. An approximate data statistics had revealed that it accounts for less than 1% of total breast malignancies having attributed for about two cases per million women annually [1,2]. They are mostly encountered in women suffering from Li-Fraumeni syndrome [3]. Phyllodes tumours of the breast have a propensity to transform into a breast sarcoma on recurrences following surgery but at an unusually low incidence, therefore, there is a paucity of published literature and knowledge; it is restricted to few retrospective case reviews and reports which compelled us to present the following case of conversion. Phyllodes tumour with sarcomatous differentiation run an extremely aggressive course, therefore, delay in its clinical diagnosis and treatment approach leads to an unfavourable prognosis.

## Case presentation

This is a case of a 55-year-old postmenopausal woman who was initially subjected to lumpectomy in 1994; the histopathology showed features of fibro adenoma. In 2011, she again felt a lump at the scar site which was excised having histopathological features of benign phyllodes tumour. Subsequently, from same site in 2014 and 2015, recurrences were excised showing borderline phyllodes (8 x 4 x 3.5 cm) and malignant phyllodes (3.5 x 3 x 1 cm) respectively, both with closest margin of 0.1 cm.

**Figure 1:**
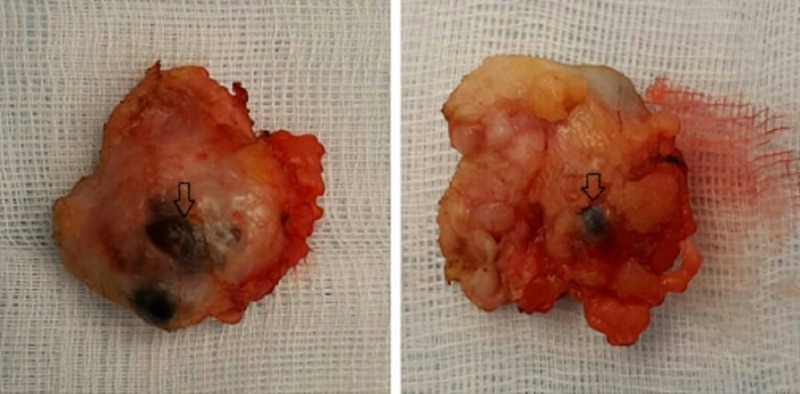
Lumpectomy specimen showing a well-circumscribed, rounded mass with areas of haemorrhage (arrows)

**Figure 2:**
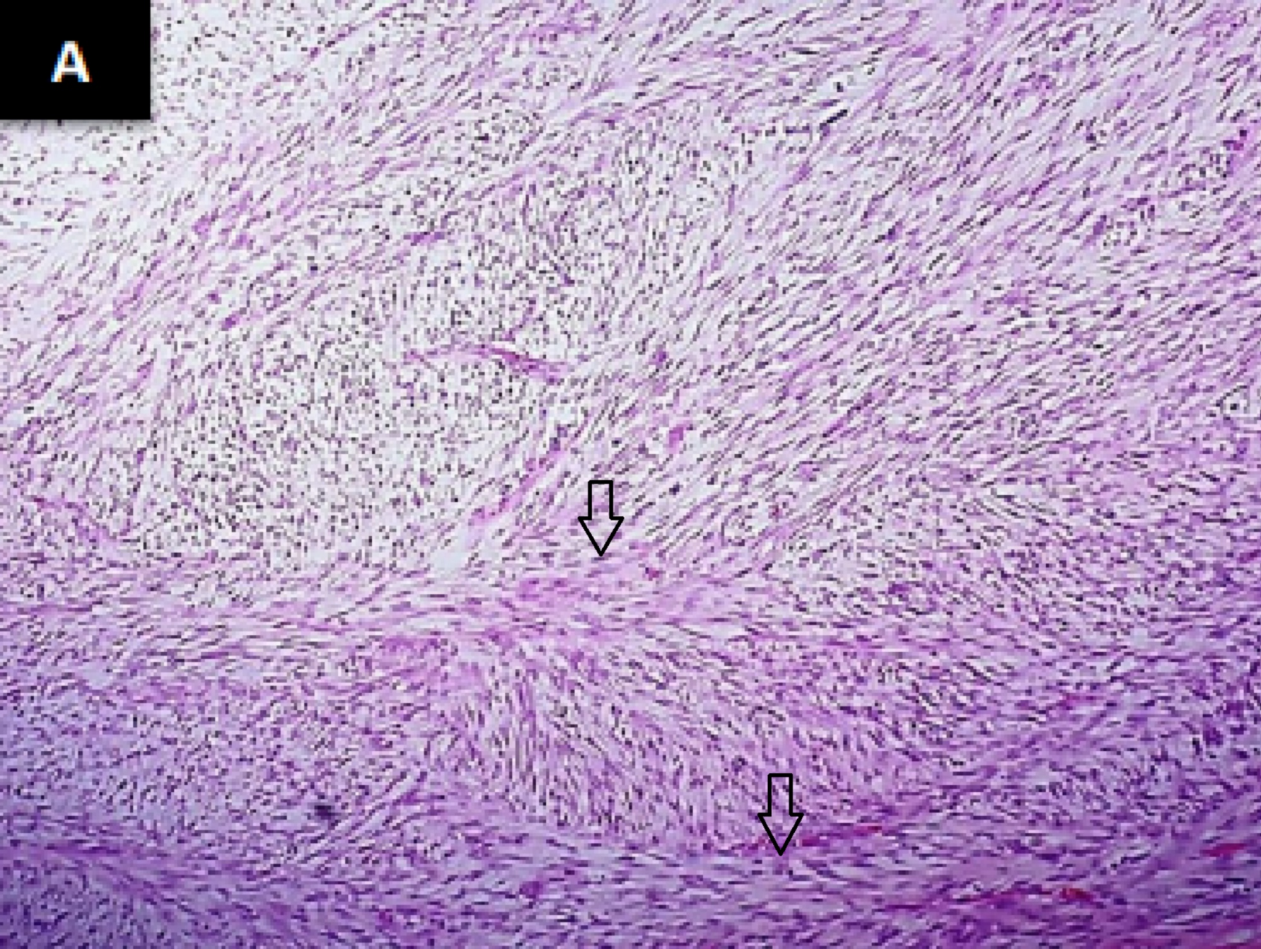
Spindle tumour cells arranged in a herring bone pattern as indicated by arrows (haematoxylin & eosin stain)

Partial mastectomy was performed in 2016; histopathological findings were consistent with malignant phyllodes of 12 x 10 x 8.5 cm with closest painted margin of 0.2 cm. Following that, computed tomography (CT) scan of the chest, abdomen, and pelvis, as well as a bone scan, was performed which were unremarkable. After three months of partial mastectomy, she underwent total mastectomy showing evidence of low-grade fibrosarcoma (1.8 x 1.7 cm) with closest margin of 1 cm (Figure 1).The previous pathological blocks were reviewed again by two senior pathologists and findings were consistent with low-grade fibrosarcoma. Sections showed the framework of the tumour was comprised of spindle cells exhibiting hyper and hypo cellular areas. These were arrayed in loose interlacing fascicles. Individual neoplastic cells had moderately pleomorphic, hyperchromatic nuclei, inconspicuous nucleoli and moderate cytoplasm. At places, herring bone pattern was appreciated and stroma was myxoid (Figure 2). Mitotic count was 20/10 high power field (HPF) and no necrosis nor epithelial component was identified. The cells of the lesion were infiltrating skeletal muscle bundles.

**Figure 3:**
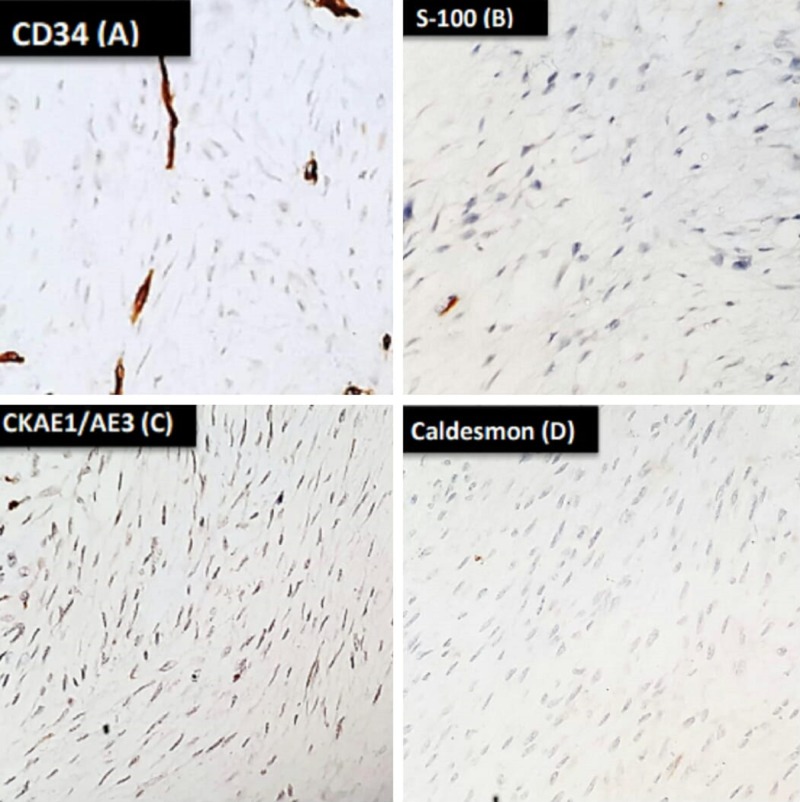
Immunohistochemical stains revealed tumour cells negative for CD34 (A), S-100 (B), CKAE1/AE3 (C), and caldesmon (D) CD:cluster of differentiation; CKAE1/AE3: cytokeratin AE1/AE3.

Immunohistochemistry was carried out with the following immunohistochemical markers to exclude malignant phyllodes tumour and metaplastic carcinoma: cluster of differentiation (CD)34, S-100, cytokeratin (CK) AE1/3, and caldesmon (Figure 3). All were negative in spindle cells supporting the diagnosis of breast fibrosarcoma. Later on, she was subjected to chest wall radiation with boost total 7000 Gy. She remained quite well for 1.5 years; subsequently, she developed three tiny nodules at the scar site, the largest was 1 cm (recurrences) which was widely excised with latissimus dorsi flap coverage. In 2018, she developed bony metastasis and pulmonary nodules with local recurrence under the flap. She was treated with metronomic chemotherapy (adriamycin, cyclophosphamide); however, she succumbed to death after four months of distant metastasis. 

## Discussion

Phyllodes tumours formerly known as serocystic disease of Brodie or cystosarcoma phyllodes have a predilection to be found in women who are above 40 years [2]. These fibroepithelial tumours, firstly described by Chelius in 1827, constitute 2%-3% of all fibroepithelial tumours [4]. On the basis of histological features, phyllodes tumours are stratified into three main varieties as benign, borderline, and malignant. Fortunately, a significant proportion of phyllodes tumours (60.0%-75.0%), encountered in clinical practice, are benign which are generally cured by performing wide local excision and carries a lower local recurrence rate of about 10.0%-20.0% [1].

The natural behaviour of phyllodes tumour is an interesting phenomenon; recurrence rates are high like other stromal tumours, especially if subjected to incomplete excision, having positive margins/or less aggressive surgical treatment. Recurrence may show higher histologic grade with increase mitosis and higher potential for distant metastasis [5]. It is also worth mentioning that reported recurrence rates are high in malignant phyllodes if their size is less than or equal to 4 cm; this was postulated because smaller tumours are subject to less aggressive surgical treatment [6].

Earlier published studies have shown that 28%-44% of local recurrence in benign phyllodes tumour present with higher histologic grades than the primary tumour, likewise borderline phyllodes can show recurrence ranges from 14%-25% and transformation to malignant phyllodes are seen in 12%-54% of recurrences [5,7].

Malignant phyllodes tumour have a completely different nature in comparison to the other two types having aggressive behaviour and a much higher rate of local recurrence reported from 23%-30% [7]. Having said that, when malignant phyllodes recur, it would show a wide spectrum of type of recurrences that could be benign in 0%-28% of cases; however, malignant recurrence is the most common (71%-100%) [5]. An interesting fact among recurrences is that the atypical changes within the stromal component can transform into sarcomatous features [8].

In this case, fibrosarcoma has been observed on recurrence of a recurrent phyllodes tumour which is known to be the most common pattern of sarcomatous transformation, the second being myxoliposarcoma. Others include chondrosarcoma, osteosarcoma, and leiomyosarcoma [8]. Contrary to the other two reported cases, our patient developed an aggressive transformation after multiple recurrences while in the case report by Gunasekaran et al., the patient developed fibrosarcoma in the very first recurrence after surgery. In the other case reported by Pant et al., the patient developed such change at the third recurrence [9,10].

Distant metastasis is rare in phyllodes tumour having a propensity for haematogenous spread especially to the lungs, liver, and bones ranging from 9.0% - 27.0%. The treatment options are limited; very few chemotherapeutic agents worked against these tumours with poor success rate and worse outcome [1]. Literature review documented a median survival of four to seventeen months with metastatic disease [11].

Treatment of phyllodes tumour is a challenging situation for multidisciplinary teams including surgeons and medical and radiation oncologists. The rare nature of disease results in the availability of only retrospective data including case reports and/or case series. Lack of prospective data or trials pose a challenge among surgeons and oncologists to offer the best possible treatment option to patients especially those who recurred, metastasized or both.

## Conclusions

Transformation from phyllodes tumour to fibrosarcoma of the breast is a rare phenomenon warranting accurate and timely diagnosis. These tumours are notorious for multiple recurrences likewise other sarcomas. There is a paucity of data supporting the role of radiotherapy and systemic chemotherapy in this rare disease to prevent recurrences and systemic spread. In the case of metastatic disease, none of the cytotoxic or immunotherapeutic agents has proven its efficacy.
